# Becoming through simulation‐based learning: Lived experiences of occupational therapy students from culturally and linguistically diverse backgrounds

**DOI:** 10.1111/1440-1630.70044

**Published:** 2025-08-21

**Authors:** Luocheng Zhang, Roma Forbes, Freyr Patterson, Adriana Penman

**Affiliations:** ^1^ School of Health and Rehabilitation Sciences The University of Queensland Brisbane Queensland Australia

**Keywords:** CALD, education, inclusive teaching, international students, occupational therapy, qualitative research, simulation, student experiences

## Abstract

**Introduction:**

With increasing diversity in health professionals education and ongoing workforce shortages, students from culturally and linguistically diverse (CALD) backgrounds form a growing proportion of occupational therapy cohorts in Australia. Simulation‐based learning (SBL) has been widely integrated as a pedagogical strategy to support students' preparation for clinical practice. However, few studies have explored the lived experiences of Australian occupational therapy students from CALD backgrounds participating in high‐fidelity SBL. This study aimed to address this gap.

**Methods:**

A qualitative descriptive study was conducted using semi‐structured interviews with 15 occupational therapy students from CALD backgrounds enrolled across Australian universities who had participated in high‐fidelity SBL. Reflexive thematic analysis was employed to identify key patterns within the data.

**Results:**

Four themes were generated: (1) between fear and growth – the emotional and psychological responses to SBL; (2) challenges in speaking up and being heard – facing language barriers in SBL; (3) balancing cultural identity – managing cultural differences in SBL; and (4) wanting to belong – navigating relationships with peers and educators in SBL.

**Consumer and Community Involvement:**

No consumers or community members were involved as research team members or in an advisory capacity in this study.

**Conclusion:**

High‐fidelity SBL provides valuable learning opportunities and also presents challenges for occupational therapy students from CALD backgrounds. Acknowledging both their struggles and strengths in SBL can help develop more tailored educational strategies. Supporting their success in SBL is crucial for creating more inclusive learning environments and advancing a more culturally competent occupational therapy workforce.

## PLAIN LANGUAGE SUMMARY

More students from culturally and linguistically diverse (CALD) backgrounds are now studying occupational therapy in Australia. Universities often use simulation‐based learning (SBL) in occupational therapy programmes. SBL aims to help students practise their skills typically before working with real patients. This study spoke with 15 students from CALD backgrounds who had taken part in SBL with simulated patients. The students talked about both the good parts and the hard parts. Many said SBL helped them grow, feel more confident and learn useful skills. But some found SBL stressful. They found group discussions hard to join. Some felt their teachers did not support them well. Language and cultural differences also made learning harder. This study shares ideas to make SBL better for students from CALD backgrounds.

**Key Points for Occupational Therapy**

- Occupational therapy students from CALD backgrounds experience both growth and unique challenges when participating in SBL.
- The factors influencing participation in SBL are multifaceted and interrelated.
- Targeted strategies are needed to enhance the inclusivity and effectiveness of SBL for students from CALD backgrounds.

## 1 INTRODUCTION

With the rapidly expanding health‐care sector and a global shortage of health‐care workers (World Health Organisation, 2018), high‐income countries, including Australia, are seeing increased enrolment of students from culturally and linguistically diverse (CALD) backgrounds in health professional programmes (Davey, 2023). Occupational therapy is no exception. Although more recent data are limited, estimates from 2016 suggest that students from CALD backgrounds make up approximately 5–20% of total enrolments in Australian occupational therapy education programmes (Yu et al., 2017), reflecting the increasing diversity among occupational therapy students. A report by Occupational Therapy Australia (2023) further highlights an anticipated 7.1% increase in demand for occupational therapists across Australia from 2021 to 2026. These trends indicate the importance of supporting and expanding a diverse student cohort to build a sustainable Australian occupational therapy workforce.

In addition to addressing workforce needs, students from CALD backgrounds contribute a rich array of health practices, cultural beliefs and perspectives to occupational therapy education (Gilligan & Outram, 2012). This diversity enhances learning environments and promotes the development of cultural competence among all students (Baker et al., 2024). However, while acknowledging these benefits, it is also important to recognise that students from CALD backgrounds often encounter a range of challenges. Research indicates that they face language and communication barriers, difficulties adapting to cultural differences, limited understanding of local health‐care systems, experiences of social segregation and even discrimination (Gilligan & Outram, 2012; Jeong et al., 2011; Korhonen et al., 2019; Lim et al., 2016). These challenges are particularly evident during clinical placements, where international occupational therapy students have been reported to experience higher rates of failure than their domestic peers in Australia (Nicola‐Richmond et al., 2017).

Entry‐level occupational therapy students must complete a minimum of 1000 hours of practice education to be eligible for professional registration (World Federation of Occupational Therapists, 2016). While various strategies have been proposed to support students from CALD backgrounds during placements, research highlights the critical role of pre‐placement preparation (Imms et al., 2018). Simulation‐based learning (SBL) has been widely recommended as an effective approach to increase preplacement readiness and, in some cases, as a partial substitute for clinical placements in health professional programmes, such as nursing, radiography, physiotherapy, speech pathology and occupational therapy (Hill et al., 2020; Imms et al., 2018; Ketterer et al., 2020; Pauli & Hughesdon, 2024; Watson et al., 2012).

SBL aims to provide experiential learning opportunities in a controlled environment where students engage in realistic clinical scenarios (Lateef, 2010). Simulation offers a safe space to develop clinical skills, particularly for situations or caseloads that may be more difficult to access or encounter in placements, where learning opportunities are often shaped by the immediate demands and priorities of patient care (Lateef, 2010; Penman et al., 2021). Previous studies have examined the benefits of SBL for students from CALD backgrounds, suggesting that SBL can help those students navigate language barriers (King et al., 2017; Rossiter et al., 2023), improve history‐taking and health assessment skills (Zhang et al., 2019), and increase confidence and cultural competence (Rossiter et al., 2023). However, several barriers may hinder their full participation, including linguistic and cultural challenges, inadequate preparation and support, and difficulties with reflective learning (Adedokun et al., 2022; Graham & Atz, 2015; Rossiter et al., 2023; Slingsby et al., 2010). There are different forms of SBL. In occupational therapy education, SBL activities are typically designed to be high‐fidelity, replicating real‐world practice and emphasising occupational identity, interviewing, and observation skills (Grant et al., 2021). According to the guidelines of the Occupational Therapy Council of Australia (2013), up to 200 hours of well‐designed SBL can be counted towards students' required practice education hours, provided that these experiences align with placement objectives and involve simulated patient interactions. Therefore, in the current study, high‐fidelity SBL specifically refers to SBL activities involving simulated patients, where simulated patients are individuals who are trained to portray a patient and take on a role to guide a simulation to meet the learning outcomes (Churchouse & McCafferty, 2012).

While research has explored SBL among health professional students from CALD backgrounds, a recent scoping review revealed that no studies have specifically investigated the experiences of occupational therapy students from CALD backgrounds in SBL (Zhang et al., 2024). Given occupational therapy's distinct learning requirements and professional competencies, there is a pressing need to understand how occupational therapy students from CALD backgrounds experience high‐fidelity SBL. Addressing this gap will inform the development of high‐quality, tailored SBL experiences that better support the learning needs of students from CALD backgrounds. Therefore, this study aims to answer the following question: What are the lived experiences of occupational therapy students from CALD backgrounds in Australia participating in high‐fidelity SBL activities?

## 2 METHODS

### 2.1 Study design

This study employed a qualitative descriptive design and conducted reflexive thematic analysis using an inductive approach (Braun & Clarke, 2022) to develop a rich understanding of a phenomenon where little prior research exists. Descriptive qualitative methods (Nayar & Stanley, 2024) were chosen as an exploratory approach to enable an in‐depth examination of the lived experiences of occupational therapy students from CALD backgrounds in high‐fidelity SBL environments. Ethical approval for the study was granted by the Human Research Ethics Committee of The University of Queensland (Approval number: 2024/HE001684). All participants provided informed consent before participating in the study.

### 2.2 Participants

A purposive sampling approach with a snowballing method was employed to recruit eligible participants across Australia. Participants were required to (1) be current occupational therapy students enrolled in an entry‐level Australian occupational therapy programme, (2) self‐identify as being from a CALD background and (3) have participated in high‐fidelity SBL activities within the preceding 6 months to maximise recall accuracy. An online recruitment survey was designed by the research team and hosted on Qualtrics (https://www.qualtrics.com) for potential participants to express their interest, confirm eligibility and provide contact information. Terminology related to entry‐level, CALD and high‐fidelity SBL was explained on the survey page to ensure clarity. The participants were also informed that they would receive a $50 (AUD) voucher as a token of appreciation for completing the interview.

Emails with recruitment information were sent to publicly available contact details of Australian entry‐level occupational therapy programme directors, requesting them to distribute the survey, along with a flyer, to their occupational therapy cohorts. The study was also advertised on the Occupational Therapy Australia Survey webpage, the lead researcher's university faculty research participant recruitment website, and various social media platforms (e.g. Facebook, WeChat). Additionally, the participants were encouraged to reach further eligible students through referrals.

After 2 weeks of survey dissemination, a follow‐up email was sent to respondents to reconfirm their interest and eligibility. A total of 43 participants confirmed their willingness to participate. To ensure a representative sample, stratified purposive sampling was employed, and a final group of 15 participants was selected proportionally based on their places of birth.

### 2.3 Data collection

An interview guide was developed by the research team, informed by the current literature and research questions, to facilitate an in‐depth discussion of students' lived experiences of participating in high‐fidelity SBL activities. The interview guide was piloted with two graduate occupational therapy students from CALD backgrounds who had experience with SBL; however, they were not included as study participants. Individual semi‐structured interviews (45–60 minutes) were conducted in November 2024, via Zoom (2024) (Version 6.2.5) with participants' permission. The interviews were audio‐recorded and transcribed by the lead researcher (L.Z.). See Table 1 for sample interview questions.

**TABLE 1:** Example interview questions and prompts.

Question	Prompt
What would you consider your home country and what is the main language spoken at home?	
Could you describe the SBL activities involving simulated patients you have participated in?	
How would you describe your overall experience in those simulation activities?	How did participating in simulation activities impact how you feel about your skills or abilities? Can you describe the emotions you experienced before, during and after the simulation activities? What do you think caused these feelings? When you were in a simulation, what roles did you feel most comfortable in? And why? Can you share any examples in simulation when you were hesitant to participate?
How do you think your language or cultural background affected your experience in simulation activities?	Can you describe anytime during the simulation when you felt uncomfortable or out of place because of language or cultural differences? If yes, can you describe what happened? What strengths did you feel that you brought to your simulation as someone from a CALD background?

### 2.4 Data analysis

Data were analysed using reflexive thematic analysis, a method that is well‐suited for identifying, analysing and reporting patterns within qualitative data (Braun & Clarke, 2022). The study followed the six‐phase approach outlined by Braun and Clarke (2022): (1) familiarising with the data, (2) generating initial codes, (3) searching for themes, (4) reviewing themes, (5) defining and naming themes, and (6) producing the final report.

Data sufficiency was determined through ongoing analysis using Braun and Clarke's reflexive thematic analysis approach (Braun & Clarke, 2022). During the final five interviews, no new themes emerged, indicating thematic saturation. As a result, data collection was concluded. The interview transcripts were then exported into Microsoft Word, and analysis began with the first author re‐watching the video recordings while conducting an in‐depth reading of the transcripts to ensure familiarity with the content and context of participants' responses. Initial coding was conducted iteratively, with the first author identifying and categorising significant features of the data relevant to the research questions. Codes were refined throughout the process as patterns and relationships emerged. Once coding was completed, the data were organised into potential themes, which were then systematically reviewed and refined to ensure coherence and alignment with the dataset. Themes were defined and named based on their relevance to the research aim of the study, with representative participant quotes noted for each theme. The preliminary theme names, descriptions and supporting quotes were presented to members of the research team (A.P., R.F.) for deliberation, refinement and subsequent agreement. To enhance analytical rigour, another author (A.P.) randomly selected transcripts and revisited the quotes, codes, and themes to verify their coherence and relevance, and any discrepancies were discussed in team meetings. The first author then drafted an initial version of the findings, with all the authors contributing to subsequent revisions. Throughout this process, the first author consistently referred to the original dataset to ensure that refinements in theme terminology and supporting quotations remained reflective of the full breadth of the data.

### 2.5 Research team, reflexivity and positioning

L.Z., the lead researcher, is a PhD candidate who draws from both personal and academic experience. As a former occupational therapy student from a CALD background at an Australian university, L.Z. has a deep understanding of the unique challenges faced by students from CALD backgrounds in occupational therapy education. Having personally navigated a new academic system, along with language and cultural challenges, L.Z. is particularly sensitive to the needs of these students and has a strong desire to advocate on their behalf. SBL, as a significant part of the occupational therapy curriculum, had a lasting impact on L.Z.'s student journey. While L.Z. appreciates the importance of SBL and recognises its inherent challenges, it became clear that the voices of students from CALD backgrounds in SBL must be heard. L.Z. seeks to better understand how occupational therapy students from CALD backgrounds learn through SBL. Ultimately, L.Z. aims not only to improve SBL itself but also to use it as a bridge to better support occupational therapy students from CALD backgrounds in their educational journey. The supervisory team consists of R.F., a physiotherapist from Aotearoa New Zealand; F.P., an occupational therapist from Australia; and A.P., a speech pathologist from Australia. All three are non‐CALD university academic staff with extensive clinical and academic teaching experience, enabling a well‐rounded perspective on allied health education. They are all experienced in designing and implementing SBL, including supporting students from CALD backgrounds during SBL, and thus were able to offer insight as SBL experts and educators. Most importantly, despite being non‐CALD, they are committed to understanding and addressing the challenges faced by students from CALD backgrounds. These researchers appreciated the opportunity to support the lead researcher in investigating this important topic.

To mitigate potential preconceptions, the lead researcher (L.Z.) maintained a reflexive journal throughout the study. Before conducting the interviews and starting the data analysis, L.Z. documented personal views and assumptions as a former occupational therapy student from a CALD background. This journaling process continued throughout the study to capture emerging insights and decision‐making rationales. Discussions on potential biases and their influence on data collection and analysis were regularly held during supervisory team meetings.

## 3 FINDINGS

Fifteen occupational therapy students from CALD backgrounds were interviewed. Most participants were female and in their mid‐to‐late 20s, with fewer students in younger or older age brackets. The majority were born in China, including Hong Kong and mainland China, while others came from Malaysia, Indonesia and Singapore. Their linguistic backgrounds varied, with many speaking Mandarin or Cantonese at home, while some reported multilingual proficiency, including English. The participants were enrolled in entry‐level bachelor's or master's programmes at nine Australian universities across six states. Specific universities have not been identified to maintain participant and university anonymity. Most participants had SBL activities at different points throughout their programmes, rather than within a single semester or year level. Additional demographic characteristics are shown in Table 2.

**TABLE 2:** Participant demographics.

Participant number	Age range	Gender	Place of birth	Language spoken at home	Degree	Year of study
1	25–34	Female	Mainland China	Mandarin	Master's	Second year
2	25–34	Male	Hong Kong	Cantonese	Master's	First year
3	25–34	Female	Mainland China	Cantonese and Mandarin	Bachelor's	Third year
4	25–34	Male	Mainland China	Mandarin and Cantonese	Master's	Second year
5	25–34	Female	Mainland China	Mandarin	Bachelor's	Third year
6	25–34	Female	Malaysia	Mandarin and English	Master's	Second year
7	35–44	Female	Hong Kong	Cantonese	Master's	First year
8	25–34	Female	Mainland China	Mandarin	Master's	Second year
9	18–24	Female	Indonesia	English, Indonesian, some Mandarin	Master's	Second year
10	35–44	Female	Hong Kong	Cantonese	Bachelor's	Fourth year
11	25–34	Female	Mainland China	Mandarin	Master's	Second year
12	18–24	Female	Hong Kong	Cantonese and Mandarin	Bachelor's	Third year
13	25–34	Female	Hong Kong	Cantonese	Bachelor's	Third year
14	18–24	Female	Singapore	English and Mandarin	Bachelor's	Third year
15	25–34	Female	Mainland China	Cantonese and Mandarin	Master's	First year

### 3.1 Themes

Four themes were generated following the analysis (1) between fear and growth – the emotional and psychological responses to SBL, (2) challenges in speaking up and being heard – facing language barriers in SBL, (3) balancing cultural identity – managing cultural differences in SBL and (4) wanting to belong – navigating relationships with peers and educators in SBL.

#### 3.1.1 Theme 1: Between fear and growth – the emotional and psychological responses to SBL

The participants described a range of emotional and psychological changes throughout SBL, including increased pre‐simulation anxiety and pressure, particularly during their first simulation experience, *‘Like before the simulation, even though it wasn't real…I was nervous … I think that was my first simulation and talking to, like, people who are not … my classmates, so it's like an unfamiliar person’* (OT Student 10).

During the preparation stage, the participants reported experiencing increased mental fatigue in an English‐speaking environment compared with their native language, primarily because of language barriers, and thus required extra preparation time. The participants needed to more carefully plan their communication, research unfamiliar terminology and rehearse clinical instructions to reduce the impact of language barriers on their performance. One participant illustrated this additional effort by highlighting the need to pre‐learn even fundamental procedural terms before engaging in simulation activities, *‘When explaining something in English, I have to have a look at… the guidance or online first, about the steps. Like some words that could be difficult, like tying the shoelaces or using—or how to wear the shoes. I need to, like, learn about the different parts of the shoes, like the tongues or toes, like that, something like that. But in Cantonese… I just need to explain directly to the client. So, yeah, so this is quite difficult for me’* (OT student 2).

The participants also described a conflict between their cultural emphasis on thorough preparation and the unpredictable nature of occupational therapy interactions with simulated patients during SBL, *‘I think we should prepare very well before we do something. But, you know, when you communicate with anyone, the situation you cannot control… as OT you needed to… interact very quickly… you cannot make a… 100% preparation… in our culture, I think many people, if you cannot prepare very well, you may feel very nervous, and… feel loss of control…’* (OT student 11).

During SBL, the expectation to contribute actively in real time often resulted in heightened emotional distress. Several participants described feeling overwhelmed, especially when they had difficulty processing information quickly enough to participate effectively. One student shared intense emotional distress in the first simulation, *‘I even cried the first time. It (simulation) was very fast‐paced; everyone had to take turns. I just blanked out—like, what should I say? I couldn't catch the flow’* (OT student 8). In such settings, students often felt they must perform to meet the expectations of simulation, which added a layer of stress that was especially challenging for students from CALD backgrounds.

The participants' stress and anxiety levels were also influenced by simulation scenarios themselves and assigned roles. One participant described increased stress when facing challenging simulated patients, noting that these patients, which were often controlled to present specific challenges, added more pressure, ‘…*when the simulated patients are like, they're grumpy, they could be grumpy and they might be really sad, and ended up crying … like, it absolutely, like, added stress on how I, or like, myself [managed the situation]*’ (OT Student 12). The participants reported feeling more at ease when their roles involved delivering prepared recommendations rather than engaging in more unpredictable tasks, such as spontaneous clinical conversations, *‘I think it was, I guess, looking for recommendations. That part, I was more comfortable. Yeah, because after already knowing the clients and, yeah, knowing their needs and what their workplace is like … ’* (OT Student 5).

For some participants, the emotional impact of the simulation extended beyond the session itself. These participants began to question their competence as future occupational therapists, particularly when they felt that they had underperformed compared with their local peers, *‘Well, because, as I said, I was usually quite overwhelmed afterwards. So I would second guess myself, and like I would have self‐doubt on like oh, is OT really something I'm capable of doing?’* (OT Student 12).

Despite the psychological stress experienced throughout SBL, most participants recognised both personal and professional growth through their participation in these activities. The participants described how the authenticity of simulation not only helped them develop clinical skills and confidence but also enabled them to identify areas for improvement and self‐reflection, ‘*… it (simulation) gave me some improvement or elevated my confidence in treating the client. So actually, self‐efficacy…it's increased after that series of simulations*’ (OT Student 7). *‘After that, I actually felt like I had learned something. Like, I felt a sense of achievement because I kind of knew what areas I needed to improve and what areas I was really good at’* (OT Student 13).

The sense of accomplishment was particularly strong following positive SBL experiences and feedback. One participant expressed an increased willingness to engage in future simulations, ‘*… after that simulation‐based learning, I've become more open towards other kinds of simulation‐based learning*’ (OT Student 6). Another participant shared how the experience pushed them beyond their usual boundaries by choosing to work with local classmates rather than relying on familiar social groups, ‘*Brave—especially for the second one. I thought I briefly stepped out of my safety zone. You know, that was really cool. I tried to work with local classmates rather than always staying with other Chinese classmates*’ (OT Student 8). The participants also described how the opportunity to reflect and receive immediate feedback in SBL helped them develop a mindset of continuous learning, *‘You always have to think about how to improve, think about how you did. I think over time, like, over the last three years, I've really learned to embrace it and actually reflect and want to hear feedback from other people’* (OT Student 14).

#### 3.1.2 Theme 2: Challenges in speaking up and being heard – facing language barriers in SBL

One of the most prevalent experiences in participating in SBL was navigating language barriers. The participants described experiencing delayed response times in communication because of the internal translation process between their native language and English, which increased their level of cognitive strain during SBL and made real‐time interactions more difficult.

*‘When something comes up in my mind, I need to process, like, okay, you provided this answer to me. In my head, like, I would just instantly think about it in Cantonese. And I would think, like, okay, what question should I ask next in Cantonese? Then, I need to … translate in my head, like, translate to English’* (OT Student 10).

The participants expressed frustration that their communication difficulties were sometimes not necessarily because of a lack of knowledge but rather to the additional time needed for language processing during SBL activities. As one student expressed, *‘… because of language … I just felt bad that I wasn't able to express myself completely …’* (OT Student 10). The participants described language barriers in terms of pronunciation differences, unfamiliar slang and varying accents. One participant reflected, *‘For older people* (SBL actors), *I couldn't really understand their accents or what they're saying. So it took me all, like, a longer processing time to realise what they're saying’* (OT Student 9). This challenge became more evident when simulated patients portrayed communication difficulties as part of their role. For example, a participant recalled difficulty communicating effectively with a simulated patient exhibiting impulsive behaviours because of a brain injury: *‘We have a client … because of his brain injury, he acts very like impulsively and during that simulation, we had to do a kitchen assessment with him …*. *And I think we found it challenging in a way that it is hard to communicate with the patient…’* (OT Student 12). In addition to difficulties understanding simulated patients, the participants also experienced challenges in being understood by others because of pronunciation differences, *‘… just the pronunciation of some words when I was conducting the cognitive assessment. And they (simulated patients) don't understand what I'm saying because I pronounced it differently … then it comes off as frustration or, like, agitation’* (OT Student 14).

The participants described an internal conflict between wanting to contribute to SBL and fearing making mistakes, as they were worried that any mistake could make them appear less competent and result in judgement, *‘… I felt embarrassed because I felt like, ‘Oh, my God! Like, am I … just saying something in the proper way, or … showing that I didn't have that pile of knowledge … That made me feel even … I want to contribute something, I want to say something, but, no, maybe it's wrong …’* (OT Student 10). Several students admitted that they hesitated to ask for clarification during SBL, as they were concerned that it might disrupt the flow of the session or draw unwanted attention to their language difficulties. One participant stated, *‘Sometimes when the patients say something like, maybe use some slang, I couldn't understand. So I don't get the meaning … And I also feel that if I ask, what does that mean. Does it sound professional? Yeah, so I can only try to pretend that I understand. But actually, I don't’* (OT Student 3). The participants also expressed concerns about how their language barriers impacted communication dynamics during SBL, not only from their own but also from the simulated patient's perspectives. One participant was worried that simulated patients might adjust their responses based on perceived language proficiency, which potentially limited the depth of communication: *‘I'm worried that … because if patients know that we are … we are not good at English, sometimes they will not express themselves more openly or … or just use some simple language. I'm not sure … they really expressed their demands clearly’* (OT Student 11).

#### 3.1.3 Theme 3: Balancing cultural identity – managing cultural differences in SBL

Many participants reported difficulties understanding the implicit social expectations in Australian health‐care settings replicated in the simulation environment. They realised that, compared with their home countries, where health‐care communication may be more structured and task‐oriented, the simulated Australian context placed greater emphasis on rapport‐building, informal conversations and flexibility. One participant expressed this confusion,*’… I would say, that's cultural difference probably—because some foreigners may prefer to have some small talk first, or, like, to chit‐chat about some kind of topics that is not related to what we are doing today’* (OT Student 2). Another participant noted, *‘I think in our culture we are the problem‐solver people. How do we help you is just to help you to solve the problems.’ Yeah, but…They (in Australia) say that sometimes to … let us … just build a good rapport …’* (OT Student 11).

Although participants expressed an eagerness to build rapport with simulated patients, they often found it challenging because of their unfamiliarity with cultural references during SBL, *‘…For example, you know, if they start talking about how—how they like to garden, how they often go to these places, which are places and activities I definitely didn't know about …”* (OT Student 9). Because of different cultural contexts and lived experiences, the participants described how this affected their clinical reasoning during SBL, ‘*Asian people are more collective in values compared to Australians' individual values … But for Australian people, they're more about being independent and relying on their family less … when that value comes in, I will think, “Oh yeah, if they're living with their husband or daughter or family member, I think they will still help them with some activities. However, that individual still wants to do that occupation by themselves …’* (OT Student 4).

Despite these challenges, the participants recognised that their cultural backgrounds offered strengths such as cultural sensitivity and awareness, which helped them build rapport more easily with simulated patients who were also from CALD backgrounds, *‘(When) I interact with patients from other CALD backgrounds, I find it easier to understand their experiences, feelings, and language barriers. I think that's my strength. Is that called resonance—a feeling of resonance?’* (OT Student 8). Another participant reflected on the value of their cultural background in fostering empathy with simulated patients, *‘…Because in Chinese culture, we always need to fight for something because we are in a very competitive environment … we can understand that struggle in their mind and mood. But some local people cannot understand that … But I think, maybe in the Australian context … The government will provide many benefits …’* (OT Student 11).

#### 3.1.4 Theme 4: Wanting to belong – navigating relationships with peers and educators in SBL

One of the key challenges reported by participants in SBL was the difficulty of working in groups with a majority of local students. The participants expressed feeling less confident and less capable in comparison, as local students tended to take on dominant roles in discussions, *‘…I feel like when working with local students, they may like (to) dominate more in the discussion. I don't know. I feel like local students is like more capable. And like compared with like international students, maybe it's because their language skills are better. So, yeah, when they're sharing, they seem more brave or more willing to share their ideas*’ (OT Student 3). Some participants also described feeling excluded in group discussions in SBL because they needed extra time to articulate their thoughts in English, *‘Sometimes they just skip my turns … Maybe I speak too slow, like I need time to process. And they just cannot—they just—they just could not wait for me to process….’* (OT Student 15). As a result, many reported adopting passive roles in SBL, *‘… And if working with local students, I feel like they can just talk all days, so I don't have to talk much. So basically, I will be an observer …’* (OT Student 4). In addition, the participants reflected on the difficulty of forming deeper peer relationships with local students beyond the context of simulations, *‘The only time we interact was during simulations when we were in a group … or you know, the only thing we talk about is during the assessment … So, and we never really have any experiences outside…’* (OT Student 9). In contrast, they consistently reported stronger psychological connections with peers from similar cultural and linguistic backgrounds in SBL, *‘*…*of course, feel much better … we are all international students or non‐native speaker. We will more understand each other when we make a mistake. Understand, like English is not our first language, and they were more patient to listen’* (OT Student 10). The participants also reported increased opportunities to contribute during SBL when grouped with other peers from CALD backgrounds, *‘Luckily, the majority (of my group members) are foreigners, so I can express myself more confidently’* (OT Student 7). *‘With international students, I can still get a chance to talk’* (OT Student 4). Despite these challenges, the participants expressed a strong desire to belong and connect with local peers in SBL, *‘I want to integrate into the local society, and although it may be many difficulties if the group were all local, I feel like I may be less confident, but at least I could learn more from them if there were more locals’* (OT Student 7).

Concerns were also raised about the bias of SBL evaluation, with participants feeling that direct comparisons with local students who were often more fluent and culturally aligned, might influence educators' perceptions, *‘So I mean that the grading to us may be biased. The difference of grading for us may be quite big when comparing with the locals … because all the things are happening in the same room … So they observe us, and at the same time, they have a chance to observe the locals, and they will see the difference between international students and local students’* (Student 2). Some felt that the additional challenges they faced were not fully recognised or supported by educators, *‘…they (educators) usually just ignore. They usually … I don't know why. Because I really struggle. I really struggle, but they just don't get me’* (OT Student 15). However, the participants felt more understood by educators from CALD backgrounds who demonstrated cultural sensitivity and adapted their communication styles, *‘I have one tutor that from Iran … when we talk, she's very aware of slow down speed, speaking speed. And she has told the other tutors that we are international students. We use a secondary language, so please give them more understanding’* (OT Student 1).

## 4 DISCUSSION

This study explored the lived experiences of occupational therapy students from CALD backgrounds in Australia, participating in SBL activities involving simulated patients. Four key themes were generated following the analysis (1) between fear and growth – the emotional and psychological responses to SBL, (2) challenges in speaking up and being heard – facing language barriers in SBL, (3) balancing cultural identity – managing cultural differences in SBL and (4) wanting to belong – navigating relationships with peers and educators in SBL.

The ‘fear and growth’ theme highlights the dual psychological experience of students from CALD backgrounds participating in SBL. The participants first described a range of challenging emotional responses, including stress, anxiety of feeling underprepared and post‐simulation self‐doubt. Previous studies involving medical and nursing students, although not specifically focussed on CALD backgrounds, have also reported significant levels of stress during high‐fidelity SBL activities (Barbadoro et al., 2023; Foronda et al., 2013). The increased stress is often attributed to the real‐time response demands and rapid decision‐making requirements inherent in high‐fidelity SBL, compared to more didactic, structured learning activities (Carrero‐Planells et al., 2021). Moderate, well‐managed stress in SBL can be beneficial, as it may enhance focus, attention and memory (Bong et al., 2016). In contrast, Bong et al. (2016) have also pointed out that excessive stress may overwhelm cognitive resources, impair the ability to retain and apply knowledge effectively and trigger unexpected emotional responses. This was reflected in the current study, where one participant cried during an SBL session because of uncontrollable stress. A randomised controlled trial involving final‐year Australian physiotherapy students has also reported that higher stress levels during SBL activities are associated with poorer learning outcomes (Cavaleri et al., 2023). The authors have further revealed that stress levels are associated with the complexity of simulation scenarios (Cavaleri et al., 2023), a finding consistent with the present study, where participants reported varying stress responses depending on the behaviour of simulated patients. Importantly, for students from CALD backgrounds, these psychological challenges can be even greater because of the need to navigate an additional layer of linguistic and cultural differences. Increased cognitive load among health professional students from CALD backgrounds participating in SBL has been well documented in previous literature (Adedokun et al., 2022; Graham & Atz, 2015). Therefore, when working with students from CALD backgrounds in SBL, educators must carefully evaluate additional sources of stress and thoughtfully structure SBL environment that balances challenge with psychological safety.

In addition, some participants described that the unpredictability inherent in SBL further increased their stress, as they often felt underprepared. This was partly because of the additional effort required to prepare for SBL, especially when they felt linguistically unready to respond to unexpected situations. These challenges may also reflect the influence of Confucian Heritage Culture, where students from East Asian backgrounds typically prefer structured, teacher‐guided learning with step‐by‐step instruction that allows for thorough preparation before performance (Chan, 2019). This was evident in the current study, where participants reported feeling more at ease in roles that involved structured tasks such as reading out prepared recommendations to simulated patients, rather than engaging in spontaneous interactions. Such Confucian values may create greater tension between students' expectations for structured preparation and the dynamic, real‐time demands of occupational therapy practice. Therefore, gradually introducing unpredictable elements into SBL activities may help students develop adaptability and flexibility, which are crucial professional skills in occupational therapy practice particularly in fast‐paced and unpredictable health‐care environments (Krusen, 2012). For example, educators may consider introducing changes in simulated patients' behaviours or adjusting details of the case scenario during SBL activities to integrate aspects of unpredictability.

Despite experiencing psychological distress, the participants described a gradual shift from fear to growth as they continued to engage in SBL activities. Many reflected that, particularly when perceived as successful, SBL helped them develop clinical skills, build confidence, become more open to feedback and identify areas for improvement. Several participants also shared that SBL experiences encouraged them to step outside their cultural and social comfort zones. These perceived benefits are consistent with the findings of a recent scoping review, which reported positive learning outcomes among health professional students from CALD participating in SBL (Zhang et al., 2024). The progression from fear to growth reflects the occupational therapy concept of becoming (Hitch & Pepin, 2021), as participants developed and transformed through meaningful engagement in SBL and their evolving professional identities as future occupational therapists.

To better understand these emotional responses, the self‐efficacy theory (Bandura, 1997) offers a useful lens. This theory explains that individuals' belief in their ability to meet challenges and succeed in a specific task influences how they regulate emotions and perform (Bandura, 1997). For students from CALD backgrounds, self‐efficacy can be negatively impacted by factors such as language barriers and cultural differences. This reduced self‐efficacy, in turn, may prevent them from participating actively or taking risks during SBL activities. One participant even expressed doubt about their capacity to become an occupational therapist. In contrast, the participants who experienced success in simulations reported a greater willingness to engage in future SBL activities. Therefore, supporting the development of self‐efficacy is critical. SBL should incorporate structured and progressive experiences that begin with low‐stakes activities to help students gradually build confidence. It is also recommended that educators provide constructive and encouraging feedback to help reduce stress and support students in reframing emotional challenges as meaningful and integral parts of the learning process.

‘Struggling to speak and be heard’ was a dominant finding that participants felt influenced their engagement in SBL. The participants described challenges such as internal translation processes, slower response times, pronunciation difficulties, and unfamiliarity with accents and medical terminology, all of which impacted their ability to communicate effectively with simulated patients. This finding aligns with previous research which has identified linguistic barriers as a significant challenge for health professionals students from CALD backgrounds in SBL (Zhang et al., 2024). In occupational therapy, where therapeutic communication is foundational (Fan et al., 2022), language‐related barriers may prevent students from demonstrating their full clinical capabilities. As one participant reflected that their difficulties during SBL were not because of gaps in clinical reasoning, but were instead the result of challenges in articulating their thoughts in English. Therefore, educators should be mindful that communication difficulties experienced by students from CALD backgrounds during SBL may not reflect actual deficiencies in their clinical reasoning skills, but rather difficulties in expressing knowledge in a second language. To ensure that students can be heard, it is recommended that debriefing sessions include opportunities for students to clarify their reasoning, especially when language may have limited their performance during the simulation. In addition, providing targeted language support strategies such as introducing common clinical terminology prior to simulation, offering peer practice opportunities and scaffolding communication tasks can help students better prepare for the linguistic demands of SBL.

Some participants hesitated to ask for clarification because of concerns about appearing unprofessional, particularly when lacking confidence in their language proficiency. Previous studies have similarly reported that allied health, including occupational therapy students from Asian backgrounds, often face challenges with asking questions, influenced by cultural norms that emphasise listening to educators over actively engaging with or questioning them (Lim et al., 2016; Newton et al., 2023). This reluctance to speak may also be linked to Asian ‘face saving’ tradition, where individuals maintain social harmony and avoid making mistakes or causing embarrassment to themselves or others (Prutskikh & Merkulova, 2022). The fear of interrupting a session or exposing linguistic struggles suggests a lack of psychological safety, which contrasts to the intended design of SBL as a supportive and low‐risk learning environment (Lateef, 2010). Therefore, it is critical to foster a psychologically safe environment where students from CALD backgrounds feel comfortable asking questions and fully participating without judgement or shame.

The participants in the current study also expressed concerns that simulated patients may simplify their language or limit their informational expression based on perceived student language proficiency. As interaction is very much a two‐way process, such modifications may compromise the fidelity of the simulation and may not accurately reflect the complexities of real‐world therapeutic encounters. However, as the findings were based solely on student perspectives, future research could explore how simulated patients perceive and respond to students from CALD backgrounds, particularly in relation to language proficiency and communication style. When addressing language‐related challenges, educators must critically reflect on what constitutes reasonable accommodations in SBL to facilitate student engagement while maintaining the fidelity of the simulation experience.

The theme of ‘balancing cultural identity’ highlights the complex cultural negotiations required of occupational therapy students from CALD backgrounds during SBL. A key challenge for these students lies in understanding simulated Australian health‐care norms. Many participants in this study originated from health‐care cultures typically characterised as task‐oriented and hierarchical, with formal and directive communication style (Claramita & Pratidina Susilo, 2024). In comparison, Australian clinical practice emphasises rapport‐building, informal conversations and reduced hierarchy (English et al., 2022; Yates et al., 2016). This cultural contrast created uncertainty for some participants as they attempted to balance their culturally shaped understanding of professional communication with the expectations of the simulated Australian health‐care environment.

The participants in the current study also identified specific cultural differences that influenced their participation in occupational therapy SBL. Some reported unfamiliarity with ‘typical occupations’ that Australian simulated patients focussed on, as well as environmental aspects such as housing structures, both of which are central to occupational therapy's person–environment–occupation–performance model (Baum et al., 2015). Moreover, the participants described how their cultural beliefs regarding independence and family involvement in patient care influenced their clinical reasoning in SBL. A central philosophy of occupational therapy is to promote independence (Schell et al., 2013). However, people from collectivist cultures, such as those from Asian backgrounds, often place greater value on interdependence and family roles in care (Mehta & Leng, 2006). This contrasts with Australia's more individualistic culture that prioritises autonomy and self‐reliance (Dolan et al., 2019). This cultural difference may impact how students interpret simulated patients' goals and expectations in SBL. These culturally embedded references and beliefs are integral to delivering client‐centred care and should be explicitly introduced and discussed in SBL to better support students from CALD backgrounds.

Despite cultural challenges, some participants recognised the strengths their diverse backgrounds brought to SBL. These included an enhanced ability to build rapport and demonstrate empathy towards simulated patients from CALD backgrounds, as well as offering unique perspectives on occupational therapy interventions. This aligns with findings from Yu et al. (2017), who argued that international occupational therapy students are a valuable asset to the profession, as they provide intercultural learning opportunities that enhance cultural competence among peers and educators. While much of the existing literature has focussed primarily on the challenges faced by health professionals students from CALD backgrounds in SBL, this study highlights how their cultural backgrounds can also be a source of strength that should be recognised and embraced. At the same time, this perspective aligns with a growing shift in higher education towards inclusive practices that support cultural diversity, rather than expecting students from CALD backgrounds to simply adapt and assimilate (Arthur, 2017).

Another critical aspect of the findings is the impact of peer and educator relationships on students' experiences in SBL. The participants frequently reported challenges in group simulations as they required additional time to process information and formulate responses. However, local students seemed unaware of these needs and tended to dominate group interactions. This dynamic resulted in some participants in the current study adopting passive roles in SBL, which in turn raised concerns about being seen as less competent than their local peers. Similarly, previous studies have reported that health professional students from CALD backgrounds often experience difficulties connecting with local peers and fear being looked down upon or perceived as underperformers in SBL settings (Adedokun et al., 2022; Graham & Atz's, 2015). The participants in the current study also reported feeling excluded from group discussions and experiencing a sense of marginalisation. Despite these challenges, many were eager to connect with local peers and valued opportunities for cross‐cultural change. However, this aspiration is often hindered by relational and communicative barriers, and occupational therapy students from CALD backgrounds frequently struggle to develop a strong sense of belonging within health professions education settings (Yu et al., 2023).

Furthermore, the participants expressed frustration when educators failed to acknowledge their additional challenges, which further reinforced their feelings of being overlooked. In contrast, when the participants were placed in culturally diverse groups or interacted with educators who demonstrated cultural sensitivity, they reported feeling more comfortable and included. This finding is consistent with previous research that nursing students from CALD backgrounds view peers and educators with similar cultural backgrounds as strong sources of support in SBL (Adedokun et al., 2022). Therefore, it is important to design SBL environments that foster inclusive peer interactions and culturally responsive educator–student relationships, helping students from CALD backgrounds feel a stronger sense of belonging and engagement.

### 4.1 Limitations and future directions

This study has several limitations. First, while the sample included participants from various Asian backgrounds, other cultural groups were not represented. Second, most participants were female, which resulted in a gender imbalance in the sample. However, it is reflective of the current gender distribution in the occupational therapy profession, where females constitute the majority of the workforce. Finally, as all the interviews were conducted in English, the participants' language proficiency may have influenced their ability to fully articulate their thoughts and experiences. This may have affected the depth and richness of the data. To mitigate this limitation, interviews were conducted in a conversational and supportive manner, with prompts and opportunities for clarification throughout.

While this study provides valuable qualitative insights into students' experiences, further research using diverse methodologies, such as quantitative or mixed methods approaches, could explore the relationships between linguistic and cultural factors, simulation experiences, and educational outcomes. As this study did not include the perspectives of educators or simulated patients, future research incorporating these perspectives could provide a more comprehensive understanding of cultural and linguistic dynamics in SBL. It would also be beneficial to conduct longitudinal studies to examine how simulation experiences shape students' performance during clinical placements and contribute to the development of their professional identity over time. In addition, future research should specifically examine the preparation and support needs of occupational therapy students from CALD backgrounds within SBL contexts.

## 5 CONCLUSION

This study provides important insights into the lived experiences of occupational therapy students from CALD backgrounds in Australia participating in high‐fidelity SBL. The recognition of the complex interplay among psychological, linguistic, cultural and social factors impacting engagement in SBL establishes a strong foundation for future pedagogical and curricular considerations and research in occupational therapy. The findings not only highlight the challenges that students from CALD backgrounds face but also reveal the unique strengths they bring, which collectively shape their learning experiences. As occupational therapy programmes increasingly engage diverse student cohorts and incorporate SBL into their curricula, the findings from this study contribute to the development of inclusive teaching practices and support the preparation of a culturally competent future occupational therapy workforce.

## AUTHOR CONTRIBUTIONS

All the authors contributed to this study, including all the steps. Luocheng Zhang, the first author, is a current PhD student at The University of Queensland. Freyr Patterson, Adriana Penman and Roma Forbes hold doctoral degrees and are academics at The University of Queensland. These authors provided regular supervision and guidance to Luocheng Zhang from conceptualisation to manuscript preparation. Adriana Penman conducted a review of transcripts to examine the quotes and codes to contribute to the rigour of the data analysis process. Luocheng Zhang drafted the initial version of the manuscript, and all authors contributed to its revision and finalisation. All authors have read and approved the final manuscript.

## CONFLICT OF INTEREST STATEMENT

The authors have no conflicts of interest to declare.

## Data Availability

The data supporting this study's findings are not publicly available due to ethical restrictions and the confidentiality of participants. De‐identified excerpts may be available from the corresponding author upon reasonable request and with appropriate ethics approval.
